# Impact of MWCO and Dopamine/Polyethyleneimine Concentrations on Surface Properties and Filtration Performance of Modified Membranes

**DOI:** 10.3390/membranes10090239

**Published:** 2020-09-18

**Authors:** Mariane Carolina Proner, Ingrid Ramalho Marques, Alan Ambrosi, Katia Rezzadori, Cristiane da Costa, Guilherme Zin, Marcus Vinícius Tres, Marco Di Luccio

**Affiliations:** 1 Laboratory of Membrane Processes, LABSEM, Department of Chemical and Food Engineering, Federal University of Santa Catarina, Florianópolis 88040-970, Brazil; mariproner@gmail.com (M.C.P.); ingrid_ra_malho@hotmail.com (I.R.M.); alan.ambrosi@ufsc.br (A.A.); katia.rezzadori@ufsc.br (K.R.); guilhermezin@gmail.com (G.Z.); 2 Laboratory of Control and Polymerization Processes, LCP, Department of Chemical and Food Engineering, Federal University of Santa Catarina, Florianópolis 88040-970, Brazil; cristiane.costa@ufsc.br; 3 Laboratory of Agroindustrial Processes Engineering, LAPE, Federal University of Santa Maria, Cachoeira do Sul 96508-010, Brazil

**Keywords:** membrane surface modification, antifouling, hydrophilicity, mussel-inspired, protein

## Abstract

The mussel-inspired method has been investigated to modify commercial ultrafiltration membranes to induce antifouling characteristics. Such features are essential to improve the feasibility of using membrane processes in protein recovery from waste streams, wastewater treatment, and reuse. However, some issues still need to be clarified, such as the influence of membrane pore size and the polymer concentration used in modifying the solution. The aim of the present work is to study a one-step deposition of dopamine (DA) and polyethyleneimine (PEI) on ultrafiltration membrane surfaces. The effects of different membrane molecular weight cut-offs (MWCO, 20, 30, and 50 kDa) and DA/PEI concentrations on membrane performance were assessed by surface characterization (FTIR, AFM, zeta potential, contact angle, protein adsorption) and permeation of protein solution. Results indicate that larger MWCO membranes (50 kDa) are most benefited by modification using DA and PEI. Moreover, PEI is primarily responsible for improving membrane performance in protein solution filtration. The membrane modified with 0.5:4.0 mg mL^−1^ (DA: PEI) presented a better performance in protein solution filtration, with only 15% of permeate flux drop after 2 h of filtration. The modified membrane can thus be potentially applied to the recovery of proteins from waste streams.

## 1. Introduction

Membrane separation processes (MSP) are widely applied in several industry sectors, whether directly on the production line, in the treatment of residual streams, or water recovery/treatment, for their low energy consumption, simplicity of operation, and high separation efficiency [1,2,3]. In the food industry, large volumes of protein-rich waste streams are produced, especially in animal protein and dairy processing plants, and need treatment. In this context, ultrafiltration (UF) is highlighted and commonly used as a suitable alternative to conventional methods for recovery and concentration of proteins from waste streams, and as a way to minimize water loss and up-cycle byproducts like cheese whey and other animal proteins [2,4,5,6,7,8,9]. However, the formation of a polarized layer, and fouling, is still a challenging issue in the treatment of wastewaters containing proteins, due to the high interaction that they have with the membrane surface. The fouling caused, promotes decline of the permeate flux and consequent reduction in the performance of the membranes, which have to be constantly cleaned, increasing process costs [10,11,12]. In this sense, efforts are focused on methods to minimize fouling effects [13,14,15,16,17], and membrane surface modification (MSM) is currently considered the most favorable strategy [13,18,19,20,21,22,23,24].

Among the MSM techniques, the mussel-inspired method (MI) has gained interest [25,26,27,28,29,30,31,32,33,34,35]. Based on the adhesive capacity of mussels, Lee, Dellatore, Miller, and Messersmith (2007) [36] proposed the immersion of material in a solution of dopamine (DA), with slightly alkaline pH and in the presence of oxygen (conditions for DA polymerization), to form a thin adhesive polymeric layer on the surface, known as polydopamine (PDA). The PDA exhibits chemical stability, from the interactions of the catechol group, and hydrophilic characteristics, due to the presence of the amino group [27,37,38,39,40,41,42,43,44,45,46]. As a result of the hydrophilic character and affinity with several polymers, DA is a strategy highly used in MSM to create anti-fouling characteristics; reducing the interaction of the membrane surface with various solutes, including proteins, which are usually hydrophobic, improving filtration performance, and reducing cleaning cycles [30,37,38,43,47,48,49,50,51]. As an improvement of the method, Yang et al. (2014) [52] proposed a single-step deposition of DA with a hydrophilic polymer, polyethyleneimine (PEI). According to the authors, the concomitant deposition of DA and PEI generated a cross-linked polymer chain, increasing the chemical stability and dispersing polymeric agglomerates of the PDA. Furthermore, it provided a surface with a higher hydrophilic character [52].

Later studies related to the surface modification of membranes with the deposition of DA and PEI by the mussel-inspired method also identified that modified membranes presented an antifouling property, an increase in the degree of hydrophilicity, and high water permeance. Yang et al. (2016) [26] investigated the effects of varying the amount PEI used in the modification solution, and PEI molecular weight, on the properties of the polypropylene microfiltration (MF) membrane. Xue et al. (2017) [53] used the co-deposition of DA and PEI to modify polytetrafluoroethylene MF membranes, while Lv et al. (2015) [27] carried out the deposition of DA/PEI to produce nanofiltration (NF) membranes, using a polyacrylonitrile UF membrane as support. However, most studies focus on MF membranes [25,26,38,47,54], and there are only a few reports about the modification of UF membranes by co-deposition of DA with PEI [27,55,56]. Knowledge on the effect of chemical modification on membrane performance, and about different pore size or molecular weight cut-off (MWCO) of UF membranes is also important and needed. Moreover, the effect of different concentrations of DA and PEI on UF performance, and which polymer exerts the most influence on the protein solution filtration efficiency are still unclear.

In this context, we present an extensive investigation about the impact of membrane pore size, and concentration of DA: PEI solutions, on the surface properties and on the filtration performance of modified UF membranes. The work was carried out in two parts: (i) different MWCO UF membranes (20, 30, and 50 kDa) were modified by the co-deposition of DA and PEI, using a solution containing 2 mg mL^−1^ of both species; (ii) a 50 kDa UF membrane was modified through the co-deposition of DA and PEI using solutions with different concentrations. The membrane performance was evaluated in terms of physicochemical characterizations, hydraulic performance, and bovine serum albumin (BSA) filtration.

## 2. Material and Methods

### 2.1. Material

Three commercial UF polymeric membranes were acquired from Microdyn-Nadir (Wiesbaden, Hesse, Germany). UP020, UH030, and UH050 are hydrophilic polyethersulfone (PES) membranes that support temperature up to 95 °C and a pH range from 0 to 14. MWCO are equal to 20, 30, and 50 kDa, respectively.

The solution of DA and PEI was prepared with dopamine hydrochloride, polyethyleneimine (Mw = 800 Da), and tris (hydroxymethyl) aminomethane (Tris) (Sigma-Aldrich, Saint Louis, MO, USA). The model protein used in the filtration and protein adsorption tests was bovine serum albumin (BSA; code A2153; purity >96% and molecular mass of 66 kDa), purchased from Sigma-Aldrich (Brazil). The cleaning procedures were performed with ultrapure water and 0.02% sodium hydroxide (pH 10) (P.A., Lafan, Várzea Paulista, SP, Brazil).

### 2.2. Methods

#### 2.2.1. Membrane Modification

The membranes were cut into disks (9.2 cm diameter), conditioned, and fixed in Petri dishes. The conditioning consisted of immersing the samples in ethanol (99%, Synth, Diadema, SP, Brazil) for 2 h, followed by rinsing and immersion in water (ultra-purified by reverse osmosis) for 12 h to remove preservatives and ensure the complete membrane wetting.

This study was carried out in two separate parts: the first part refers to the modification of UF membranes with different MWCO (20, 30, and 50 kDa), and with a fixed concentration of the DA/PEI solution (2 mg mL^−1^ each). In the second part, the modification of the UH050 membrane with different concentrations of DA and PEI (Table 1) was assessed. The modification solution was prepared by dissolving the DA and PEI in a Tris buffer solution (pH 8.5, 50 mM). The modification process was performed by immersing the membrane in the DA/PEI solution and shaking for 12 h in an orbital shaker (TECNAL TE-420, Piracicaba, SP, Brazil) at 50 rpm and 25 ± 1 °C. After reaching the reaction time, the membrane was rinsed with water to eliminate excess of the solution, and stored in water. The modification was carried out in duplicate, with two different membrane sheets; the reaction time (12 h) was determined in preliminary tests, and the concentration of DA and PEI (2 mg mL^−1^) was chosen based on previous works [50,54].

#### 2.2.2. Membrane Characterization

The chemical structure of the control and modified membranes was analyzed through Fourier transform infrared spectrometry, with an attenuated total reflection accessory (FTIR, model Tensor 27, Bruker Scientific LLC, Billerica, MA, USA). The polymer mass adhered to the membrane was analyzed by weighing the membrane samples (diameter 9.2 cm) before and after the modification. Before each weighing, the membranes were dried for 2 h at 40 ± 1 °C and placed in a desiccator for 1 h. The deposited polymer mass was estimated by calculating the mass difference of the samples. Membrane roughness was evaluated by surface topography with atomic force microscope (AFM) analysis (Easyscan2 Flex AFM, Nanosurf, Liestal, Switzerland), using WS × M 5.0 software (Nanosurf, Liestal, Switzerland). An electrokinetic analyzer (SurPASS, Anton-Paar GmbH, Graz, Austria) was used to determine the zeta potential of the membranes before and after modification. The analysis was carried out using a solution of KCl (1 mM), varying the pH range from 3 to 10.5 by the addition of sodium hydroxide, at room temperature (25 °C). The zeta potential of the BSA solution (2.5 g L^−1^; pH 6.5) was measured in a dynamic light scattering device, with a capillary cell and two electrodes (Zeta sizer Nano ZS3600, Malvern Instruments, Malvern, England, UK). 

Membrane wettability was evaluated by two methods. First, control and modified membranes were cut into samples of 1 cm x 2 cm and dried for 2 h at 40 ± 2 °C. In the first method (treatment 1), the samples were only immersed in water for 12 h, while in the second treatment (treatment 2), the samples were previously immersed in ethanol for 2 h, then followed by water immersion for 12 h. Immersion in ethanol in the second method was carried out to secure the total wetting of the membrane pores. This test was carried out in duplicates, and the mass water gain is given in g water/g dry membrane based on the difference in wet and dry membrane masses. The sessile drop method (Ramé–Hart, 250-F1) was used for obtaining a pure water contact angle, which was measured in five different positions on the membrane surface. The assay was performed in triplicate.

In protein adsorption assays, control and modified membranes were cut into samples of 1 cm × 2 cm and conditioned as described in the wettability tests. Then, the membranes were placed in a desiccator for 1 h, weighed, and conditioned again before being immersed in a tube with 7 mL of 0.5 g L^−1^ BSA solution at pH 6.5. The tube with the membrane and the BSA solution was shaken for 6 h in an orbital shaker (TECNAL TE-420, Piracicaba, SP, Brazil) at 130 rpm and 25 ± 1 °C. Then, protein solution aliquots were collected to determine the protein concentration by Bradford’s method. The mass of protein adsorbed to the membrane in µg protein/mg dry membrane was calculated with Equation (1).

(1) $$q=\frac{\left(C_{i}-C\right)V}{M_{d}}$$

where *M_d_* (g) is the mass of the dry membrane and *V* (L) is the volume of the solution in the tube. *C_i_* (g L^−1^) and *C* (g L^−1^) are BSA initial, and over time, concentrations, respectively.

#### 2.2.3. Filtration Performance

The water permeance and the permeate flux of the BSA solution were evaluated using a conventional stirred cell (dead-end), with a volume of 500 mL and an effective membrane area of 9.6 cm^2^. The driving force was the pressure exerted by the injection of nitrogen into the cell headspace, regulated by a digital manometer (0–5 bar). The tests were performed at 23 ± 2 °C and under agitation with a magnetic stirrer (1000 rpm). Before the filtration tests, the control membrane (unmodified) was conditioned in ethanol for 2 h and immersed in ultrapure water for 12 h. The modified membranes were used directly from the storage vessel (ultrapure water). 

The water permeance was obtained according to a procedure adapted from Zin et al. (2019) [57]. The filtration system was filled with water and pressurized at 5 bar, keeping the permeate collecting valve open until constant flux (membrane compaction), then the permeate flux was collected, varying the pressure from 4 to 1 bar. For the protein solution filtration and fouling tests, 100 mL of BSA solution (2.5 g L^−1^, pH 6.5) was used. The tests were performed under agitation (magnetic stirrer, 1000 rpm) and the constant pressure of 4 bar for 2 h. Permeate flux was obtained every 15 min. At the end of the process, samples of permeate and concentrate were collected to analyze the total protein content by the Bradford method [58], and to calculate the membrane retention. After the protein solution filtration, physical and chemical cleaning procedures were carried out in sequential steps. The physical cleaning (PC) was performed with 100 mL of ultrapure water under agitation for 10 min. Chemical cleaning (CC) was carried out with 100 mL of 0.02% sodium hydroxide solution (pH 10) for 30 min, with a solution refresh after 15 min. At the end of the CC, the membrane was washed with 100 mL of water for 5 min. All cleaning procedures were performed with the membrane coupled to the system under agitation (magnetic stirrer, 1000 rpm). After each cleaning procedure, a water permeation test was performed.

Experiments to evaluate the membrane regeneration, with the control and modified membrane, were performed with three consecutive cycles of protein filtration (2 h each), interleaved by physical cleanings. At the end of the third filtration, a complete cleaning procedure (PC + CC) was carried out, and water permeance determined after each cleaning procedure. 

### 2.3. Statistical Analysis

The results were presented as an arithmetic mean ± the standard deviation of two replicates, and the Tukey test (significance of 5%) was used to identify significant differences.

## 3. Results and Discussion

### 3.1. Influence of Membrane Molecular Weight Cut-off

The effect of different MWCOs on the membrane modification by the mussel-inspired method was assessed through testing 20 (UP020), 30 (UH030), and 50 kDa (UH050) PES UF membranes, modified with DA and PEI concentration of 2 mg mL^−1^ and 12 h of reaction time. 

#### 3.1.1. Physicochemical Characteristics of Control and Modified Membranes

**Figure 1:**
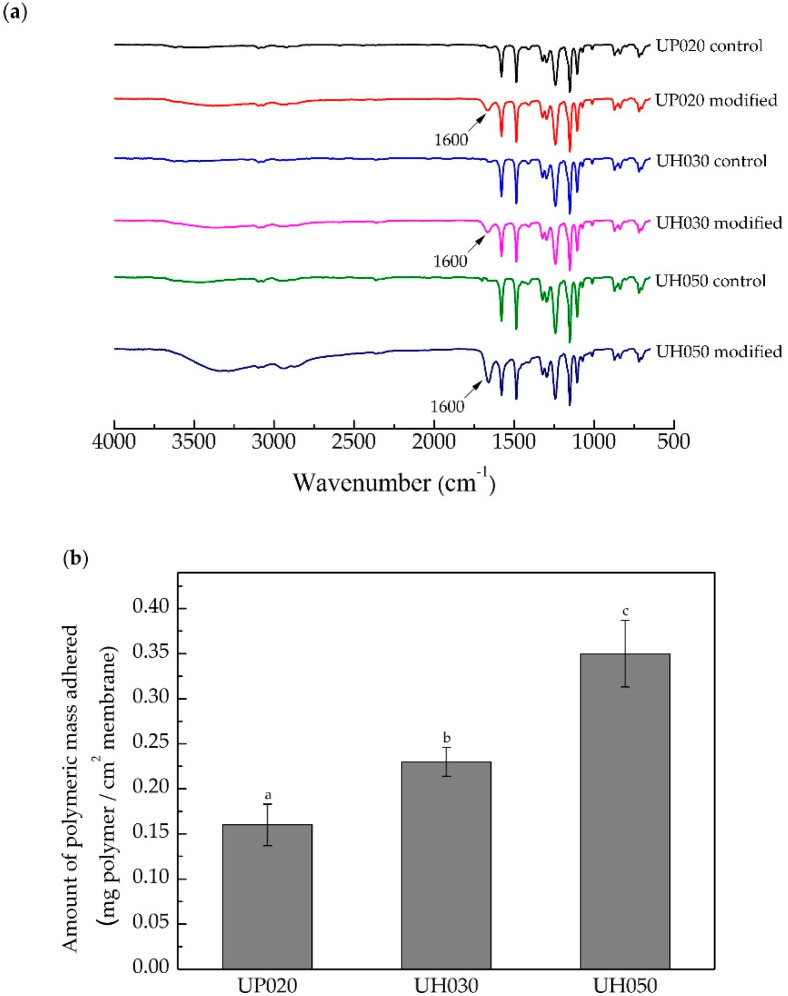

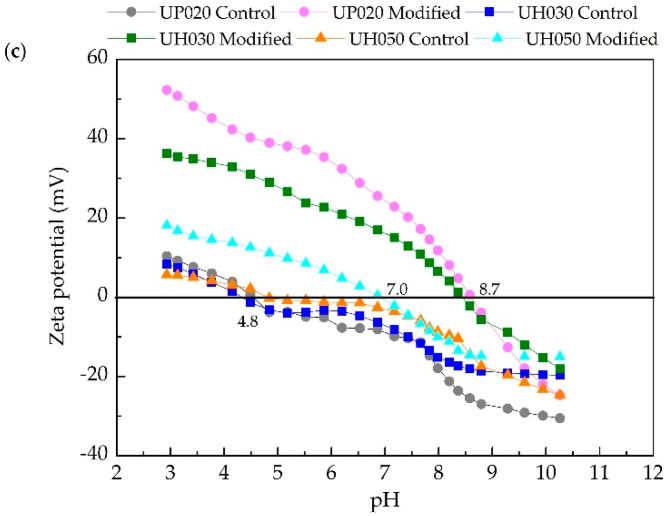
(**a**) ATR-FTIR spectra of the control and modified membranes. (**b**) Polymeric mass deposited on modified membranes with DA and PEI concentrations of 2 mg mL^−1^ and a deposition time of 12 h. Bars with different letters show the statistical difference (*p* < 0.05). (**c**) Zeta potential of control and modified membrane with DA and PEI concentrations of 2 mg mL^−1^ and deposition time of 12 h.

The chemical structure of the control and modified membranes was analyzed by FTIR-ATR to check the effective reaction of DA and PEI, which is reported to occur through a Michael addition or Schiff base reaction between the catechol and amine groups [52]. The spectra of the modified membranes (Figure 1a) shows a band between 1600 and 1630 cm^−1^, attributed to vibrations of the C = N bond [52,59]. This band suggests the primary amine (PEI) and the carbonyl group (catechol) formed a Schiff base, confirming the incorporation of PDA/PEI on the surface of the PES membrane [55,57,60]. Moreover, the larger the MWCO, the higher the amount of DA/PEI mass deposited on the membrane, as shown in Figure 1b. The UP020, UH030, and UH050 modified membranes presented a gain of polymer mass equal to 0.16, 0.23, and 0.35 mg cm^−2^, respectively, which is an indication that as the membrane pore size increases, the polymeric solution penetrates more easily into the membrane pores, which can lead, not only to the membrane surface modification, but also to changes of the inner surface of the pores. Nevertheless, the average roughness (Figure S1 of Supplementary Material) was not modified. The roughness of the UP020, UH030, and UH050 control membranes was 6.79, 4.86, 17.75 nm, while the modified ones were 6.69, 6.63, 17.87 nm, respectively.

Thus, for better comprehension of the effects of the modification on membrane properties and their relationship to MWCO, zeta potential analysis was performed. With this analysis, it is possible to gather information on the surface charges and to identify possible changes after the modification reaction. Figure 1c shows the zeta potential of the control and modified membrane surfaces within the pH range 3–10.5.

The control membranes, independently of the MWCO, have a similar distribution of charges (Figure 1c). Their isoelectric point (IP) is around pH 4.8, which shows that the membrane surface is mostly negatively charged in the evaluated pH range. The simultaneous deposition of DA/PEI on the membrane surface considerably changed the surface zeta potential, and the IP of the UP020 and UH030 membranes moved to approximately pH 8.7. In contrast, the IP of the UH050 membrane moved to pH 7.0. A more positive membrane zeta potential was expected after the modification, due to the upturn of the number of amino groups on its surface. These results confirm that the membrane modifications were successfully carried out.

The MWCO also caused variation in zeta potential after the modification process. Figure 1c shows that the modified membrane with the larger average pore size, i.e., UH050, exhibited fewer positive charges on its surface when compared with the other two modified membranes (UH030 and UH020). Such behavior implies that the smaller pore size hinders the penetration of the DA/PEI solution into the membrane pores, and the modification process tends to occur only on its surface. Thus, due to the higher number of amino groups present on its surface, the membranes with smaller pore sizes are more positively charged. This behavior corroborates the discussion related to the polymer mass adhered to the membrane, since the zeta potential is a surface analysis. In other words, the results infer that the deposition of DA/PEI on the membrane with the higher MWCO occurs both on the surface and onto the pore walls.

**Figure 2:**
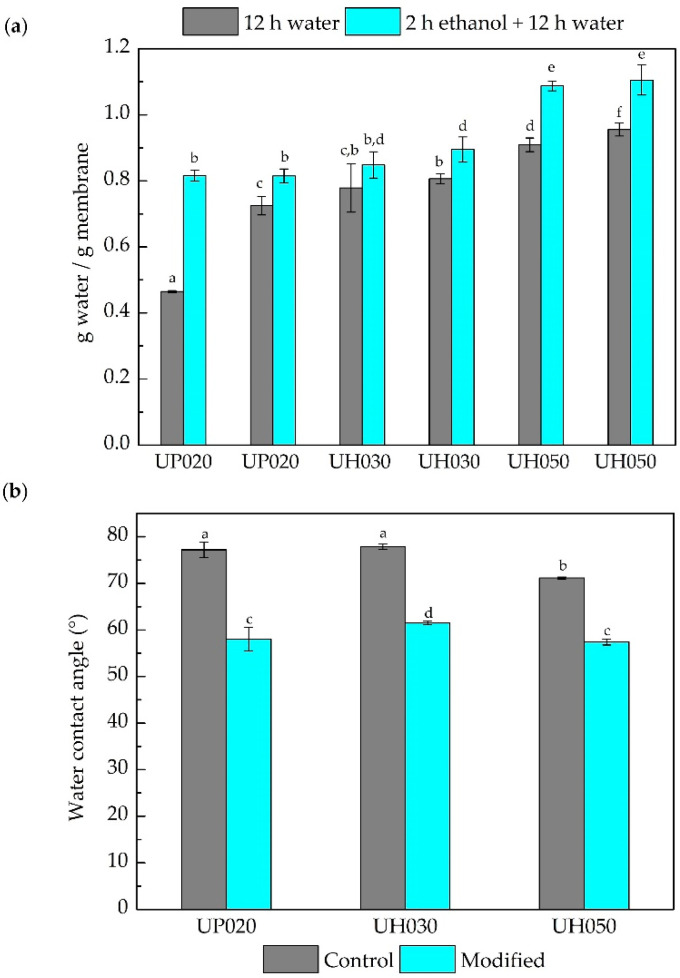
(**a**) Wettability of control and modified membranes measured after two different treatments. In the first bar, membranes were immersed in water for 12 h (treatment 1). In the second adjacent bar, the membranes were immersed in ethanol for 2 h and then immersed in water for 12 h (treatment 2). (**b**) Water contact angles for control and modified membranes. Bars with different letters show a statistical difference (*p* < 0.05).

The hydration properties (wettability and hydrophilicity) of the control and modified membranes are shown in Figure 2. The amount of water absorbed after the two treatments (treatment 1—12 h water and treatment 2—2 h ethanol + 12 h water) was similar for UH030 and UH050 control, and modified membranes, except for the UP020 membrane (Figure 2a). The larger the pore size, the more water is absorbed, which is reasonable since there is more pore volume available to be filled in with water.

The different behavior presented by the UP020 control compared to the controls UH030 and UH050 after treatment 1 (only water) can be explained by the difference in the membrane pore sizes. Since all the control membranes had a hydrophilic character, the UH030 and UH050 showed lower resistance to water absorption owing to their larger pore size. For the UP020 control, this absorption was more difficult because of the smaller pore size and the consequent higher Laplace pressure for water intrusion. However, after the treatment with ethanol, which has a lower surface tension than water (22.1 and 72.3 mN/m at 20 °C, respectively), the water can fill the membrane pores more easily. It is worth noting that wettability assay shows the amount of water that the membrane is able to retain both in the pores and absorbed in the polymer matrix. Since the UF membrane is porous, we can consider that the amount of water inside the pores is much larger than that absorbed by the polymer. Thus, the membrane can absorb a limited amount of water, which is slightly increased by the ethanol conditioning. Moreover, membranes composed of the same polymeric material, but with a larger pore size, retain more water than membranes with a smaller pore size due to capillarity.

As expected, all the membranes presented a hydrophilic character (contact angle <90°) (Figure 2b); the lower contact angle of the UH050 control membrane (~70°) may be related to its larger pore size than the other two membranes tested (~75°). Additionally, the membranes presented a decrease (~20%) in contact angle after the chemical modification, regardless of pore size. Wang et al. (2020) [3] modified PES ultrafiltration membranes with dopamine and surfactant, and obtained similar contact angle results for control and modified membranes.

**Figure 3:**
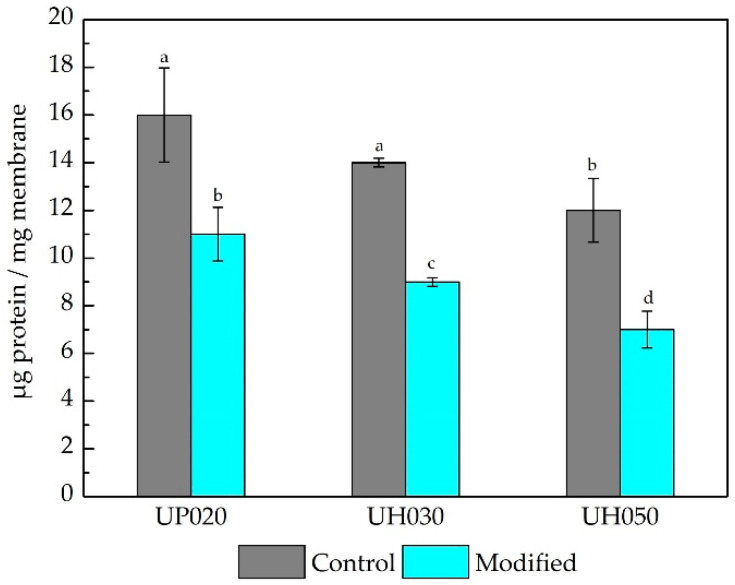
Bovine serum albumin (BSA) adsorption on the surface of control and modified membranes at a concentration of 0.5 g L^−1^ for 6 h. Bars with different letters show a statistical difference (*p* < 0.05).

The increase of the hydrophilic character was responsible for the decrease of BSA protein adsorbed on the membrane surface, as we can observe in Figure 3. All modified membranes adsorbed a lower amount of protein (31%, 36%, and 42% for the UP020, UH030, and UH050 modified membranes, respectively) compared to their controls. Likewise, the higher the MWCO, the lower the mass of protein adsorbed. This behavior matches the results presented in Figure 1b,c, which evidence that with increasing the membrane pore size, the DA/PEI solution modifies the membrane surface and the walls of the membrane pores, therefore decreasing the interaction PES/protein.

#### 3.1.2. Membrane Filtration Performance of Control and Modified Membranes

**Figure 4:**
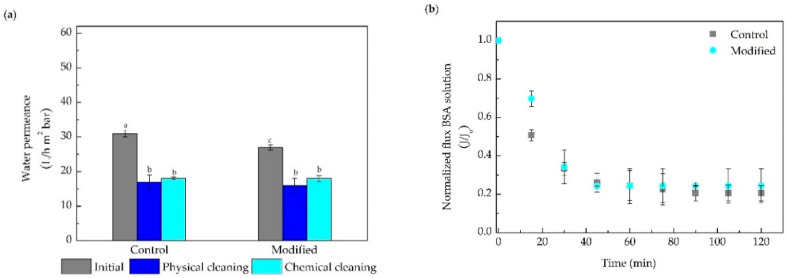

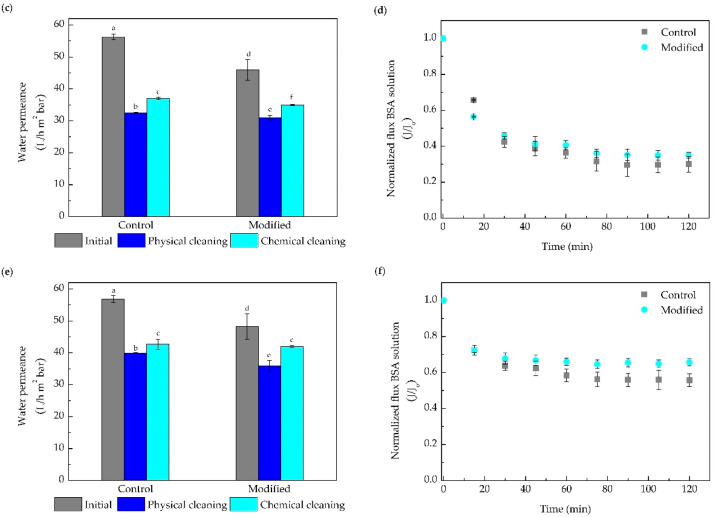
Water permeance before the protein filtration (initial) and after the physical and chemical cleaning for the membranes (**a**) UP020, (**c**) UH030, and (**e**) UH050. Normalized permeate flux of the BSA solution (**b**) UP020, (**d**) UH030, and (**f**) UH050. Bars with different letters show a statistical difference (*p* < 0.05).

The filtration performance of the modified membranes, evaluated in terms of water permeance (before protein filtration and after cleaning steps) and normalized BSA solution permeate flux is presented in Figure 4. All the membranes presented a slight reduction (10% for UH030 and UH050, and 15% for UP020) in the initial water permeance after modification (Figure 4a). The difference in the water permeance drop may be due to the larger MWCO of the UH030 and UH050 membranes, which facilitates the entrance of the DA/PEI solution into the membrane pores, and leading to a slight reduction of the membrane pore size when compared with the UH020 modified membranes. Similar behavior was observed elsewhere [27,31]. Membrane retentions were all above 98% since the membrane MWCOs were smaller than the molecular size of the BSA, which is around 65 kDa.

Despite the lower BSA adsorption presented by the modified membranes in comparison to the control (Figure 3), the normalized permeate flux of BSA solution over 2 h filtration (Figure 4b,d,f) was similar for the UP020 and UH030 membranes. On the other hand, the UH050 modified membrane showed a 20% drop in permeate flux when compared to the control membrane after 2 h of filtration. However, it is important to consider that the concentration of the solution used in the filtration tests was five times higher (2.5 g L^−1^) than that used in the protein adsorption test (0.5 g L^−1^). With a high protein concentration in the feed solution, differences are less prone to be detected by the filtration tests. The increase in MWCO induced an improvement in filtration, both in control and in the modified membrane, due to the reduction of the membrane resistance to water flux. As previously discussed, the DA/PEI solution can penetrate the pores more easily in larger pore size membranes, forming not only a thin layer on the surface but also coating the pore walls. Thus, when the aqueous protein solution comes into contact with the membrane, the water quickly bonds with the membrane surface creating a higher resistance to fouling, which is reflected in the increase in the permeate flux of the membranes with larger pore size. This behavior is supported by the results of polymeric mass adhered, zeta potential, and adsorption tests (Figure 1 and Figure 3). 

Figure 4a,c,e also show that the UP020, UH030, and UH050 control membranes recovered around 60, 66, and 73% of the initial water permeance after chemical cleaning, while the modified membranes recovered 67, 75, and 85%, respectively. Concerning MWCO, when increasing the pore size of the membrane, the recovery of water permeance increased significantly; a remarkable result pointing to a possible application in industry. The results of BSA normalized permeate flux (Figure 4b,d,f) and recovery of the water permeance after the cleaning procedures (Figure 4a,c,e) indicate that the membrane modification in larger MWCO membranes improves the protein filtration performance.

In general, the results observed indicate that the modification of the membrane surface by the co-deposition of DA/PEI is affected by MWCO. With the increase in the MWCO, the improvement in the filtration performance of the modified membrane became more evident, when compared to its control, and with a lower decline in permeate flux compared to the initial flux, and an increase in the recovery of the water flux after cleaning procedures. Thus, the UH050 membrane was chosen to proceed to Part 2 and evaluate the different DA/PEI concentrations.

### 3.2. Influence of DA and PEI Concentration 

Although the results presented in Section 3.1 indicated an improvement in the performance of protein solution filtration through UF membranes modified with DA and PEI, with more intensity of the membrane with higher MWCO, tests varying the concentration of these polymers were performed to investigate the direct influence of the polymers on the filtration performance. Initially, we sought to identify which polymer, DA or PEI, would be responsible for the greatest effect on the membrane performance when filtering protein solutions. Then, the concentration was varied.

#### 3.2.1. Effects of Different DA and PEI Concentrations

**Figure 5:**
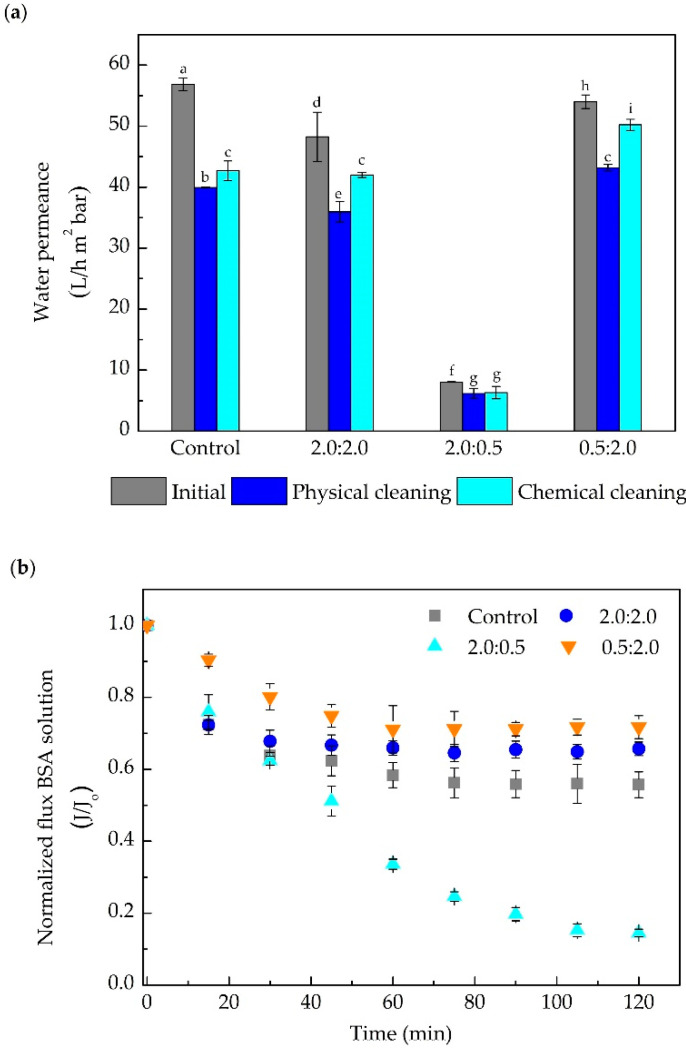
Performance of control and modified UH050 membrane with different concentrations of DA:PEI (2.0:2.0; 2.0:0.5, and 0.5:2.0 mg mL^−1^) in the filtration of BSA solution (2.5 g L^−1^). (**a**) Water permeance before the protein filtration (initial) and after the physical and chemical cleaning. (**b**) Normalized permeate flux of the BSA solution. Bars with different letters show a statistical difference (*p* < 0.05).

Different polymeric blends of DA and PEI (2.0:0.5 and 0.5:2.0 mg mL^−1^) were used to modify the membranes for 12 h, making it possible to compare with the results from Section 3.1.2 (UH050 membrane, 2.0: 2.0 mg mL^−1^ of DA/PEI). The performance of control and modified membranes in the permeation of the BSA protein solution are presented in Figure 5.

Figure 5a shows that the different polymeric blends of DA:PEI used in the modifying solution considerably interfere with the water permeance. While the modified membranes with DA:PEI concentration 2.0:2.0 and 0.5:2.0 mg mL^−1^ presented initial water permeance (before the protein filtration) close to that of the control membrane, the modified membrane with the concentration 2.0:0.5 mg mL^−1^ showed a 80% drop in the permeance. This behavior can be explained by the higher hydrophilic character of the PEI in comparison to the DA. Another fact that should be highlighted is the increase in the polymeric growth rate of the PDA film on the membrane surface when using modification solutions with a higher concentration (m/m) of DA (a good binding agent). This explanation agrees with some studies claiming that the small pore size of UF membranes can be blocked by the thicker polymeric films formed after modification with polymeric blends with higher concentrations of DA [40,50]. Moreover, Yang et al. (2014) [52] suggested that the incorporation of PEI through crosslinking with the catechol and amino groups eliminates the PDA aggregates, helping to increase the hydrophilicity of the membrane, without significantly affecting the permeate flux that could be compromised by pore blockage. Such behavior can be seen in the water permeance results obtained in this part of the study. All membranes presented retention values above 98% (data not shown), an expected result since the membrane had a molecular weight cut-off lower than the molecular size of the BSA.

Figure 5b presents the normalized flux of BSA solution for the control and modified membranes at 2 h of permeation. The normalized flux was around 20% and 35% higher than the control for the membranes modified with 2.0:2.0 and 0.5:2.0 mg mL^−1^ of DA/PEI, respectively. In turn, a normalized flux decrease of around 70% at the end of the filtration was observed for the membrane modified with 2.0:0.5 mg mL^−1^ of DA/PEI, when compared to the control. Such behavior is in agreement with the discussion concerning the water permeance, increasing the amount of PEI (a super hydrophilic polymer) reduces polymeric aggregates and enhances the interaction of the surface with water, increasing the water permeance and BSA solution permeation. 

The recovery of the water permeance after the cleaning procedure is shown in Figure 5a. Results indicate that at least 70% of the water permeance can be recovered by using only physical cleaning (pure water under stirring), as in the case of the control membrane. A gain of up to 15% can be noticed for the modified membranes, with the best result obtained by the 0.5:2.0 DA:PEI sample (80% recovery). Similar behavior is observed for the permeance recovery regarding chemical cleaning, in which the membrane modified with the highest proportion of PEI, i.e., 0.5:2.0 mg mL^−1^, was the one that presented the highest permeance recovery (93%). In turn, the control membrane showed the lowest recovery percentage after chemical cleaning (73%). These results suggest that the use of a higher amount of PEI in the modification solution decreases the solute/membrane interaction, favoring the cleaning procedure (results of the zeta-potential analysis presented in the next section prove this hypothesis). Moreover, PEI, a highly hydrophilic polymer, is mainly responsible for increasing the permeate flux and decreasing the fouling effect; the lower the DA:PEI ratio, the more promising results were obtained. To understand the extent to which reducing this ratio impacted the process, we investigated the membrane modification by increasing the PEI concentration and keeping DA concentration constant.

#### 3.2.2. Influence of PEI Concentration 

**Figure 6:**
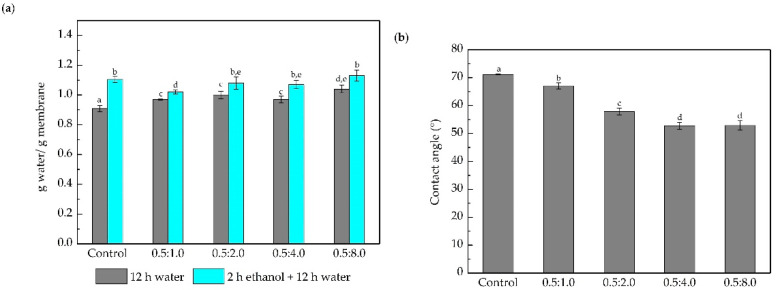
(**a**) Mass water uptake of control and membranes modified with different concentrations of DA and PEI after the immersion for 12 h in water (treatment 1), and the immersion for 2 h in ethanol and then 12 h in water (treatment 2). (**b**) Water contact angle of the control and membranes modified with different concentrations of DA and PEI. Bars with different letters show a statistical difference (*p* < 0.05).

Membrane surface modification was performed with a reaction time of 12 h and different polymeric blends of DA:PEI. The DA concentration was fixed at 0.5 mg mL^−1^ and the concentrations of PEI evaluated were 1.0, 2.0, 4.0, and 8.0 mg mL^−1^. The hydrophilicity of the membranes was evaluated by contact angle and water mass gain (Figure 6).

**Figure 7:**
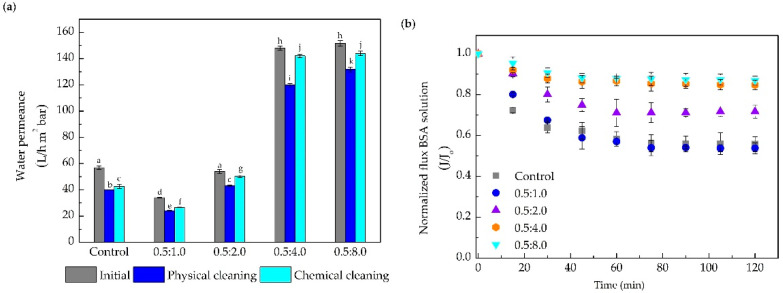
Performance of the control and modified membranes with different concentrations of DA and PEI (0.5:1.0, 0.5:2.0, 0.5:4.0, and, 0.5:8.0 mg mL^−1^) in the permeation of BSA solution (2.5 g L^−1^). (**a**) Water permeance before the protein filtration (initial) and after the physical and chemical cleaning. (**b**) Normalized permeate flux of the BSA solution. Bars with different letters show a statistical difference (*p* < 0.05).

The increase in the capacity of the modified membranes to interact with water, when compared to the control membrane is observed in water uptake results (Figure 6a). After treatment 1 (water), the water uptake slightly increased (~10%) with the PEI concentration increase. After treatment 2 (ethanol + water), it can be observed that the modified membranes present a similar water uptake to the control membrane. It is important to point out the limit of water that can be absorbed by the membrane, as explained previously (Figure 2). The increase in PEI concentration from 0.5 to 4.0 g L^−1^ caused the contact angle (Figure 6b) to decrease by about 20%. Zin et al. (2019) [57] modified a poly (vinylidene difluoride) microfiltration membrane with DA/PEI to treat oily wastewater, and observed similar behavior. According to Yang et al. (2016) [26], the increase in the degree of hydrophilicity is a result of a large number of amino groups present in the PEI molecule, and consequently in the PDA/PEI coatings. The augmentation of membrane hydrophilicity may induce a higher resistance to the adhesion of hydrophobic components on its surface, and further investigation, by evaluating the hydraulic performance of the membranes in the UF of BSA solution supported this hypothesis. The results are shown in Figure 7.

Figure 7a illustrates that the water permeance before the protein filtration (initial) varied by increasing the PEI concentration from 1.0 to 8.0 mg mL^−1^. Membranes modified with high PEI concentration presented higher permeance. Water permeance augmented by about 170%, compared to the control membrane, when using 4.0 and 8.0 mg mL^−1^. This behavior corroborates the discussion in Section 3.2.2, in which the PEI, due to its super hydrophilic characteristic, and capacity to reduce polymeric aggregates, is mainly responsible for the increase of the water permeate flux. In addition, the increase in hydrophilic groups on the membrane surface due to the DA/PEI coating facilitates the water flux through the pores, reflected in the higher permeance observed [61]. All the membranes evaluated presented the same protein retention, around 98%.

All the membranes presented a similar behavior regarding the normalized flux of BSA solution over time, as illustrated in Figure 7b. Control and modified membranes with 0.5:1.0 mg mL^−1^ of DA/PEI presented almost equal values of normalized flux, with a 45% drop at 2 h of filtration. In turn, the membranes modified with higher concentrations of DA/PEI showed a lower decline in permeate flux. For the 0.5:4.0 and 0.5:8.0 mg mL^−1^ modified membranes, a decline of only 15% was noticed, proving that the increase in the PEI concentration, in relation to DA in the modification process, is beneficial to the performance of the membrane. In addition, this result corroborates the indication that an increase in the concentration of PEI generates an increase in the affinity with water, which reduces possible interactions of solute/membrane, and is reflected in a smaller drop in flux over the filtration period. Zin et al. (2019) [57] suggested that the formed DA/PEI film provides a hydration layer, making it difficult for hydrophobic solutes to adhere into the modified membrane surface. This is also supported by the protein adsorption test (Figure S2 of Supplementary Material), in which modified membranes adsorbed 60% less BSA. This reduction in the amount of adsorbed protein corroborates that a possible increase in the number of hydrophilic groups favors the interaction of water with membrane surface, and increases resistance to adsorption of hydrophobic compounds (BSA). Other authors have also observed that membranes modified by PDA or co-deposition with other polymers adsorbed smaller amounts of protein than unmodified membranes [25,62]. 

The results presented in Figure 7a also show a better performance in terms of water permeance recovery was achieved with the membranes modified with higher concentrations of PEI. After the physical cleaning, the permeance recovery of the membranes modified with 0.5:2.0; 0.5:4.0, and 0.5:8.0 mg mL^−1^ was around 80%. On the other hand, for the control and modified membranes with a lower concentration of PEI, this recovery was around 70%. When analyzing the recoveries after the chemical cleaning step, the modified membranes with concentrations of 0.5:2.0, 0.5:4.0, and 0.5:8.0 mg mL^−1^ achieved recoveries of 93, 96, and 95%, respectively, which are excellent results for the membrane cleaning process. 

Differences were also found for the membrane zeta potential over the pH range evaluated (Figure S3 of Supplementary Material). The control membrane was predominantly negatively charged, presenting a point of zero charges (PZC) at pH 4.5. In turn, for 0.5:4.0 and 0.5:8.0 mg mL^−1^ DA/PEI membranes, the PZC was increased to pH 5.3 and 5.7, respectively. The differences among the membranes can be attributed to the increase in the number of amino groups, as discussed in item 3.1.1. Additionally, the zeta potential results help to evaluate the attraction or repulsion between the protein molecules and the membrane surfaces at pH 6.5 (filtration pH). At pH 6.5, the BSA solution showed zeta potential values equaling −8.9. Control, 0.5:4.0 and 0.5:8.0, membranes showed zeta potential values equaling −8.6, −14.4, and −17.7, respectively. Thus, as the BSA solution and membranes showed negative zeta potential values, the protein tends to be repelled when in contact with the membrane surface, thus avoiding adsorption and fouling. The modified membranes were also more negatively charged when compared to the control membranes, increasing the repulsion between the protein and the membrane surface. These results, associated with the hydrophilicity (Figure 6), corroborate the hypotheses discussed for the filtrations of the BSA solution (Figure 7) in which, PEI, a highly hydrophilic polymer, is mainly responsible for the improvement in performance of PES UF membranes modified by the present method. 

#### 3.2.3. Membrane Regeneration

**Figure 8:**
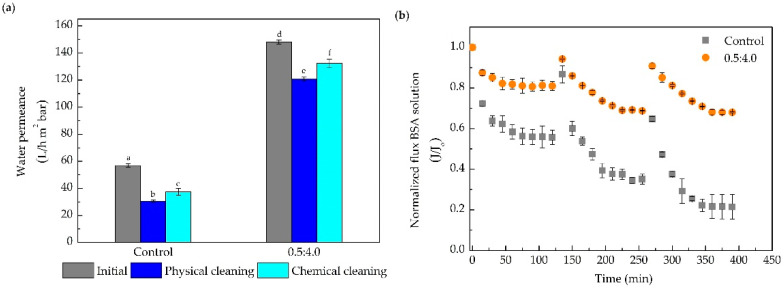
Membrane regeneration experiments of the control and modified membranes with 0.5:4.0 mg mL^−1^ of DA and PEI in the filtration of BSA solution (2.5 g L^−1^). (**a**) Water permeance before the protein filtration (initial) and after the physical and chemical cleaning (after three cycles of BSA filtration). (**b**) Normalized permeate flux of the BSA solution. Bars with different letters show a statistical difference (*p* < 0.05).

Membrane regeneration assays consisted of three consecutive cycles of protein filtration (2 h each) interleaved by physical cleanings that were also performed (Figure 8). Since no differences between the membranes modified with 4.0 and 8.0 mg mL^−1^ of PEI were observed, this assay was carried out with the control and the membrane modified with 0.5:4.0 mg mL^−1^ of DA, and PEI.

The modified membrane, which exhibited an increase in the water permeance and protein retention similar to the control (above 98%), was able to conduct the protein filtration with a decline in permeate flux (protein permeate flux) three times smaller on average. The modified membrane showed better resistance to fouling, with a reduction of only 30% of the permeate flux at the end of the third cycle, in relation to the initial flux of the first cycle (Figure 8b). On the other hand, the control membrane presented a permeate flux decline close to 80% at the end of three filtration cycles. Substantial recovery of water permeance after the cleaning procedures (Figure 8a) was obtained with a modified membrane, when compared with the control membranes (around 50% higher for the physical cleaning and 40% for the chemical cleaning). 

As a final consideration, the results presented here corroborate the assumption that modification by coating with DA/PEI can increase the affinity with water, and probably reduces the protein adsorption on the membrane surface, improving the resistance to fouling. Moreover, the choice of PEI concentration used to modify the UF membranes, through co-deposition with DA, plays an essential role in the improvement of the protein solution filtration performance.

## 4. Conclusions

This study explored the impact of membrane pore size and concentration of dopamine and polyethyleneimine (DA:PEI) solutions used to modify membrane surface and improve filtration performance, when treating wastewaters containing protein. PES ultrafiltration membranes modified by the co-deposition of DA/PEI increased the resistance to fouling in protein solution (BSA) filtration. The performance of the modification process was affected by the MWCO of the membrane and also by the DA/PEI concentration used in the modification solution. The modified membrane with larger MWCO (50 kDa) presented the lowest protein adsorption, when compared with control membranes and with UH020 and UH030 modified membranes. In addition, experimental results showed high DA concentration decreases the water permeance and the permeate flux of the protein solution. On the other hand, an increase in the PEI concentration resulted in more hydrophilic membranes and is mainly responsible for the better performance in the protein filtration process. Thus, membranes modified with 0.5:4.0 and 0.5:8.0 mg mL^−1^ of DA and PEI showed better results in BSA solution filtration and antifouling properties. 

## Figures and Tables

**Table 1:** Dopamine (DA) and polyethyleneimine (PEI) concentrations used for UH050 membrane modification.

DA (mg mL^−1^)	PEI (mg mL^−1^)
2.0	0.5, 2.0
0.5	1.0, 2.0, 4.0, 8.0

## Supplementary Materials

The following are available online at https://www.mdpi.com/2077-0375/10/9/239/s1. Figure S1: AFM images of the control and modified membranes, Figure S2: Static adsorption test of BSA on the surface of the control and modified membranes. The test was performed for 6 h with a BSA solution concentration of 0.5 g L−1. Bars with different letters show statistical difference (p < 0.05), Figure S3: Zeta potential of the control and modified membranes. The DA and PEI concentrations used to modify the membranes were 0.5:4.0 and 0.5:8.0 mg mL−1.
