# Supplementary material for: Impact of MWCO and Dopamine/Polyethyleneimine Concentrations on Surface Properties and Filtration Performance of Modified Membranes
Source: Membranes (Basel). 2020 Sep 18;10(9):239. doi: 10.3390/membranes10090239 (PMC7559832; doi:10.3390/membranes10090239)
Supplement: Supplementary file 1 [file membranes-10-00239-s001.pdf]

Supplementary material

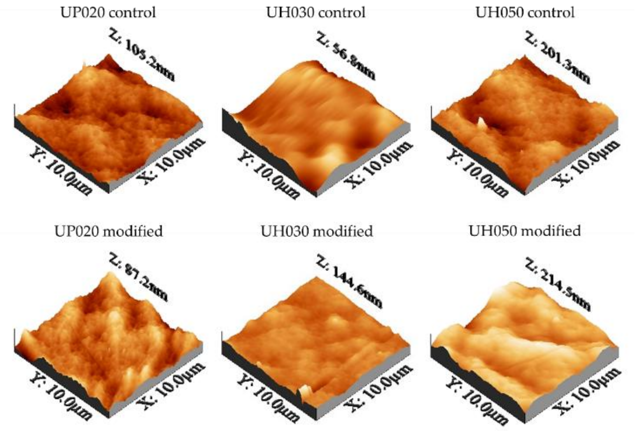

Figure 1. AFM images of the control and modified membranes.

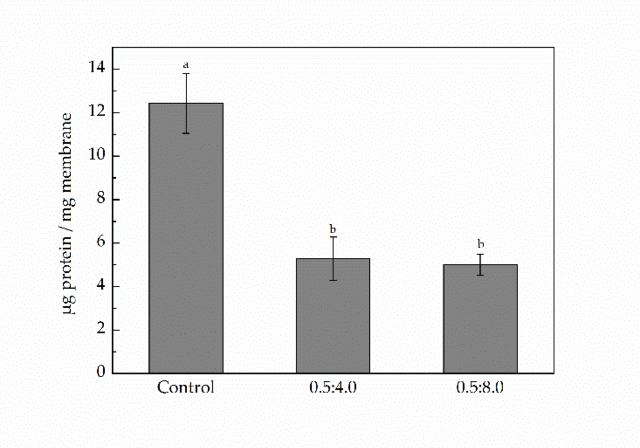

Figure S2: Static adsorption test of BSA on the surface of the control and modified membranes. The test was performed for 6 h with a BSA solution concentration of 0.5 g L^{-1}. Bars with different letters show statistical difference ($p < 0.05$).

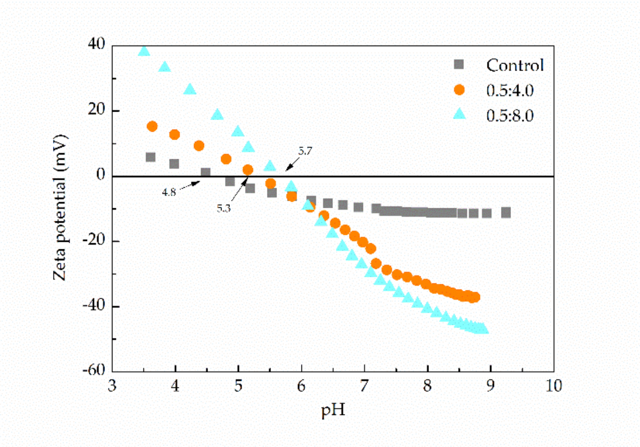

Figure S3: Zeta potential of the control and modified membranes. The DA and PEI concentrations used to modify the membranes were 0.5:4.0 and 0.5:8.0 mg mL^{-1}.
